# Timeliness of the second dose of measles-containing vaccine uptake and its determinants among children aged 24–36 months in Gondar City, Northwest Ethiopia, 2023: Community-based cross-sectional study design

**DOI:** 10.1016/j.jve.2025.100594

**Published:** 2025-03-17

**Authors:** Molalign Aligaz Adisu

**Affiliations:** Department of Pediatrics and Child Health Nursing, College of Medicine and Health Sciences, Woldia University, Woldia, Ethiopia

**Keywords:** Timeliness, Second dose, Measles, Vaccination, Ethiopia

## Abstract

**Background:**

Measles remains a global public health concern, despite the availability of effective vaccines. Recent outbreaks highlight the need for strong vaccination programs. Since launching both doses, Ethiopia has been working with global health organizations to increase vaccination coverage. However, focusing solely on coverage overlooks the importance of timely vaccination. In Ethiopia, despite occasional increases in coverage, measles outbreaks persist due to insufficient attention to timeliness. This study aims to assess the timeliness and its determinants of second-dose measles-containing vaccine uptake in Gondar City to inform efforts to strengthen immunization programs and prevent measles infections.

**Methods:**

A community-based cross-sectional study was conducted among 618 children aged 24–36 months. Participants were selected using a two-stage systematic random sampling method from April 25 to May 25. Structured questionnaires were administered through interviews, and data were collected using the Kobo toolbox and then analyzed using Stata version 17. A binary logistic regression model was utilized to determine factors associated with the outcome, with significance declared at a p-value <0.05. Adjusted odds ratios with 95 % confidence intervals were used to assess the direction and strength of associations.

**Results:**

Among the total of 618 children, 523 (84.63 %) (95 % CI: 81.77 %–87.48 %) were vaccinated for MCV2 timely (in the national recommended age). Paternal college and above in their education (AOR: 5.84, 95 % CI: 1.55–8.18), four or more ANC follow-ups (AOR: 5.84, 95 % CI: 1.55–8.18), at least two doses of vitamin An uptake (AOR: 6.39, 95 % CI: 2.92–12.59), mothers having high awareness (AOR: 2.04, 95 % CI: 1.05–3.99), and mothers having positive perception (AOR: 4.81, 95 % CI: 2.13–10.86) to measles vaccination were significant determinants for timely uptake of the second dose measles-containing vaccine.

**Conclusion and recommendations:**

The timely uptake of the second dose of the measles vaccine in the study area was suboptimal, and efforts should be continued to eradicate measles infection. Paternal educational status, ANC follow-ups, repeated vitamin An uptake, maternal awareness, and perception of measles vaccination were statistically significant determinants for the timely uptake of a second dose of measles-containing vaccine. Strengthening maternal and child health services, increasing awareness, and changing mothers' perceptions about measles vaccination may increase the timely uptake of MCV2 among children receiving a second MCV dose.

## 1 Introduction

Measles remains a significant global public health concern despite the availability of effective vaccines.^1^, ^2^, ^3^ In recent years, outbreaks of measles have been reported in various parts of the world, highlighting the importance of robust vaccination programs to control and prevent the spread of this disease. The measles-containing vaccine (MCV) is highly efficacious, with one dose providing immunity to approximately 85 % of recipients.^2^ However, to ensure long-term protection and minimize the risk of outbreaks, a second dose of measles-containing vaccine (MCV2) is recommended by the World Health Organization (WHO).^4^ When both doses are administered according to the recommended schedule, the effectiveness of measles vaccination increases significantly, providing over 95 % protection against measles infection.^5^

Ethiopia launched the first measles-containing vaccine (MCV1) in early 1981 and MCV2 in February 2019^6^ Since then, the Ethiopian government, in collaboration with international health organizations such as the World Health Organization (WHO) and the United Nations Children's Fund (UNICEF), has been working to increase vaccination coverage and reduce the burden of measles in the country, and fruitful efforts have been made to improve coverage.^6^^,^^7^ While achieving high vaccination coverage rates is essential, the timeliness of vaccination is also a critical factor in preventing the spread of measles. Timely administration of MCV2 is vital for maintaining immunity levels and preventing gaps in protection.^8^^,^^9^

The determinants that were found to be significantly associated with the timeliness of MCV2 uptake included mothers' educational level, residential location, household head, access to mass media, birth order, availability of vaccines, attendance at antenatal and postnatal care visits, and mothers' awareness of measles vaccination.^10^, ^11^, ^12^, ^13^, ^14^, ^15^ Parental perception of vaccination is also another important determinant for accepting childhood vaccination^16^

Global coverage of MCV2 has been increasing but remains relatively low. In 2020, global coverage was estimated at 71 %. In low-income countries, particularly in sub-Saharan Africa the coverage of second dose measles vaccine is low. In 2020, estimated second dose coverage in the African region was only 54 %. Coverage varies widely between countries in sub-Saharan Africa. Some countries like Rwanda, Zambia, and Botswana have achieved over 90 % coverage, while others like Chad, Central African Republic, and Equatorial Guinea have coverage below 30 %.^17^ Even though the coverage of MCV2 in Ethiopia is occasionally increasing (For example the coverage of MCV2 in north Shewa in 2022 was 42.5 %,^18^ in Gojjam 48 %,^19^ and in the study area in 2023 was 75.68 %^20^), measles outbreaks continue to become a public health concern in the country.^7^^,^^21^ This is due to the government, health authorities, and researchers having concentrated their primary focus on vaccination coverage, neglecting the timeliness of the vaccination. Without administering the measles vaccine promptly, at the recommended age, the measles outbreak will not be eliminated. The study emphasizes that improving the timeliness of measles vaccination is a key strategy for achieving and maintaining measles elimination goals. Monitoring and addressing barriers to timely vaccination is an important public health priority.^3^ In contrast, the study found that delayed or late vaccination is associated with an increased risk of measles infection and outbreaks. Leaving children vulnerable to measles at younger ages, when they are at higher risk of complications, can have serious public health consequences.^22^

In our knowledge of searching, there has been no research conducted regarding the timeliness of MCV2 uptake in the study area. Therefore, this study aims to assess the timeliness of MCV2 uptake and its determinants in Gondar City. The study helps to inform policymakers, healthcare providers, and community stakeholders in their efforts to strengthen immunization programs and protect public health from measles infection. It could be also used as a reference for further investigation into the timeliness of MCV2 uptake in Ethiopia in future studies.

## 2 Methods

### 2.1 Study design and setting

A community-based cross-sectional study design was conducted from April 17 to May 16, 2023. The study was conducted in Gondar city. The historical city of Gondar is located in the northwest of the Amhara region approximately 741 km from Addis Ababa, the capital city of Ethiopia. According to the local administrative report, it has approximately 475,000 total populations in 2022. Among this, over 43,000 were children under the age of five years. It has twenty-four (one comprehensive specialized hospital, one General hospital, eight health centers, fourteen health posts) governmental health institutions and over fifty private/business clinics. The city is structured by 6 sub-cities which contain 36 (25 urban and 11 rural) kebeles (the smallest administrative unit).^23^ All children aged 24–36 months who live in Gondar city and were vaccinated for MCV2 regardless of the time they were taken were the source of population, and those MCV2 vaccinated children aged 24–36 months with their mothers/caretakers in selected kebele of Gondar city were the study population. Seriously ill mothers or caretakers who were unable to respond and residents of the study area for less than 6 months were excluded from the study.

### 2.2 Sample size determination

The sample size was calculated by using a single population proportion formula, **n = Z^2^**_**α/2**_**∗P (1-P)/d^2^**, where n is the number of samples required, with the assumptions of a 5 % significance level (Z_α/2_ = 1.96), 5 % margin of error (d = 0.05), and p = (50 % or 0.5) since there is no study done on the timeliness of MCV2 in Ethiopia. Considering a design effect of 1.5 and a non-response rate of 10 % the final estimated sample size was:

n = Z^2^_α/2_∗P (1-P)/d^2^

= (1.96)^2^∗0.5∗(1–0.5)/ (0.05)^2^ = 384.16∗ 1.1∗1.5 = **634**

### 2.3 Sampling technique and procedure

A stratified multi-stage sampling technique was used. First, 36 kebeles are stratified into 25 urban and 11 rural kebeles. Out of the total number of kebeles, 6 from urban areas and 3 from rural areas (comprising 25 % of the total) were randomly selected using a random sampling method. The samples were proportionally allocated to each kebele based on the number of households containing children aged 24–36 months who had received MCV2. Eligible households were selected using a systematic random sampling technique, with a sampling interval of 6. To randomly select the first household for the systematic sampling, the researchers used prominent public facilities like churches, mosques, health centers, health posts, and government offices as reference points. This ensured an unbiased starting point, from which the researchers could then systematically select through predetermined intervals. In households with more than one eligible child, the youngest child was selected to reduce recall bias (refer to Supplemental Fig. 1**)**.

### 2.4 Study variables

Timeliness of MCV2 (timely uptake and untimely uptake) **was the dependent/outcome variable of this study.** Socio-demographic factors **(**child age, maternal age, maternal educational status, marital status, monthly income, family size, mass media access, occupational status of mother, head of household, and residence), maternal and child-related factors (antenatal care (ANC) and postnatal care (PNC) visits, tetanus toxoid (TT) vaccine uptake during pregnancy, birth order, place of delivery, number of children, bacilli Calmette Guerin **(**BCG), pentavalent 3, and vitamin An uptakes), health service and access related factors **(**time taken to reach the nearest facility, waiting time at vaccination center, willingness to open multi-dose vaccine for one or two children, vaccinator, and vaccine availability), awareness and perception of mothers were independent variables.

### 2.5 Operational definitions

**Timely uptake of MCV2:** The proportion of children who received the second dose of the measles vaccine (MCV2) within 4 days prior to and within 4 weeks after the nationally recommended age of 15 months in Ethiopia.^12^^,^^24^

**Untimely uptake of MCV2:** The proportion of children who did not receive MCV2 within the specified timely uptake period.

**Awareness:** Seven awareness questions were asked and scores greater than or equal to the median were considered as having high awareness.^13^^,^^20^

**Perception:** Seven perception questions were asked and scores greater than or equal to the mean were considered as having positive perception.^25^

### 2.6 Data collection tools and procedures

A structured, interviewer-administered, and pre-tested questionnaire was used to collect the data. The questionnaire was adapted from the previous studies.^10^^,^^11^^,^^18^^,^^19^^,^^26^^,^^27^ This questionnaire consists of socioeconomic and demographic factors, maternal and childhood vaccination-related factors, health access and service-related questions, and awareness and perception of mothers about measles vaccination-related questions. Information regarding the child's vaccination status (including BCG, Pentavalent 3, and Vitamin A) and the age at which MCV2 was administered was collected from vaccination cards. If a vaccination card was not available, data were gathered through interviews with mothers or caregivers. This process involved asking about the child's age during vaccination, as well as the site and route of administration. Data collection was conducted by four degree-holding nurses and supervised by two nurses with master's degrees, along with the principal investigator.

### 2.7 Data Quality control

To maintain consistency and ensure the accuracy of the data, the English version of the questionnaire was translated into Amharic and then back into English. A pre-test was conducted on 32 participants, representing 5 % of the total sample, in the Ayira kebele, which was not part of the selected kebeles. The questionnaires were examined and restructured by the inputs following the pre-test. The reliability of the questionnaire items assessing mothers' perception and awareness of the measles vaccination was evaluated using Cronbach's alpha, which yielded a value of 0.77. This score is considered an acceptable level of reliability. Two days of intensive training have been given to the supervisors and data collectors. Every day, lead investigators and supervisors adhered precisely to the data-gathering process. Before the analysis, the necessary steps were taken to prepare the data. This included identifying and handling any missing values, cleaning the data to ensure accuracy and consistency, and verifying the information through cross-checks.

### 2.8 Data processing and analysis

The Kobo toolbox application was used to collect the data, which was subsequently exported to Stata version 17 for coding, cleaning, and analysis. Descriptive statistics were employed to characterize the features of the participants. Before conducting the regression analysis, the chi-square goodness of fit test was examined for each variable, and the variance inflation factor (VIF) was calculated to assess multicollinearity among the independent variables, with the mean VIF being 3.33. The adequacy of the model was evaluated using the Hosmer and Lemeshow goodness of fit test, which yielded a p-value of 0.94. Significant variables from the bivariable analysis with a p-value of <0.2 at a 95 % confidence interval were then incorporated into the multivariate logistic regression models to control for the effect of potential confounding factors. A p-value of less than 0.05 was considered statistically significant for the test of association. The crude and adjusted odds ratios, along with their respective 95 % confidence intervals, were calculated to determine the degree of associations between the outcome and independent variables. Finally, the results are presented through the use of text, figures, and tables.

## 3 Result

### 3.1 Socio-economic and demographic characteristics of mother-child pairs

Among a total of 634 sample sizes, 618 (with a response rate of 97.5 %) mothers/caretakers who had children aged 24–36 months participated. The mean age of the mothers was 31 (±SD 4.58) years, while the median age of children was 30 months, with an interquartile range of 27–32 months. Most of the participants were orthodox in religion and married (Table 1).

**Table 1:** Socio-economic and demographic characteristics of mother-child pairs in Gondar city, Central Gondar, Northwest Ethiopia, 2023. (N = 618).

Characteristics	Categories	Frequency	Percentage
Residence	Urban	562	90.94
	Rural	56	9.06
Sex of child	Male	317	51.29
	Female	301	48.71
Age of Mothers (years)	≤25	123	19.90
	26–30	183	29.61
	31–35	185	29.94
	≥36	127	20.55
Religion	Orthodox	500	80.91
	Muslim	90	14.56
	Others	28	4.53
Maternal Educational status	Unable to read and write	61	9.87
	Primary level	167	27.02
	Secondary level	216	34.95
	College and above	174	28.16
Mother's Occupation	Housewife	351	56.80
	Farmer	14	2.27
	Privet Business	104	16.83
	Gov.t Employee	149	24.11
Marital Status	Married	515	83.33
	Others	103	16.67
Paternal Educational Status	Unable to read and write	17	2.77
	Primary level	171	27.85
	Secondary level	197	32.08
	College and above	229	37.30
Head of household	Father	324	52.43
	Mother	83	13.43
	Both	200	32.36
	Others	11	1.78
Family Size	<5	507	82.04
	≥5	111	17.96
Average Monthly Income (US$)	<100	151	24.43
	100–200	268	43.37
	>200	199	32.20
Mass media availability	Yes	526	85.11
	No	92	14.89

### 3.2 Maternal and childhood vaccination and health service-related factors

Three-fourths (74.92 %) of children were born through planned pregnancy. Four hundred eighty (77.67 %) of the mothers took the TT vaccine at least once during the pregnancy of the index child. Four hundred twenty-nine (69.42) of the children had taken vitamin A at least twice. Only 148 (23.95 %) used PNC services at least once. Two-thirds of participants 401 (64.89 %) reached within 30 min to the vaccination site by foot. One hundred eighty-two (29.45 %) of the respondents had a history of postponement or cancellation of their vaccination schedule by vaccinators or health professionals (Table 2).

**Table 2:** Maternal and childhood vaccination status and health service-related characteristics of participants in Gondar City, Central Gondar, Northwest Ethiopia, 2023. (N = 618).

Characteristics	Categories	Frequency	Percentage
Parity	One	146	23.62
	Two	251	40.61
	Three and above	221	35.76
Birth order	1st birth order	186	30.10
	2nd-4th birth order	411	66.50
	≥5th birth order	21	3.40
Pregnancy status	Planned	463	74.92
	Unplanned	155	25.08
The child lives with whom	Both parents	505	81.72
	Mothers only	87	14.08
	Fathers only	15	2.43
	Others	11	1.78
Maternal TT vaccine status	Not received	138	22.33
	At least once	480	77.67
BCG vaccine	Vaccinated	603	97.57
	No vaccinated	15	2.43
Pentavalent 3 vaccine	Vaccinated	606	98.06
	Not vaccinated	12	1.94
Vitamin A	No dose received	43	6.96
	One dose received	146	23.62
	At least two doses	429	69.42
ANC follow up	No follow-up	23	3.72
	1-3 follow up	188	30.42
	≥4 follow up	407	65.86
PNC service utilization	Not received	470	76.05
	At least once	148	23.95
Place of Delivery	Home	8	1.29
	Health center	184	29.77
	Hospital	404	65.37
	Privet/business clinics	22	3.56
Time to arrive at the nearest vaccination center on foot	≤30 min	401	64.89
	>30 min	217	35.11
Place of vaccination	Hospital	232	37.54
	Health center	351	56.80
	Health posts	15	2.43
	Outreach site	15	2.43
Waiting time for vaccination at the vaccination center	≤30 min	347	56.15
	>30 min	271	43.85
Vaccination appointments have been rescheduled or canceled	Yes	182	29.45
	No	436	70.55

### 3.3 Awareness and perception of mothers/caretakers

From the total of 618 mothers/caretakers, the mean score regarding seven awareness-related questions about measles vaccinations was 3.67 ± 1.65 SD whereas the mean perception score was 3.39 with ±1.37SD. Of all, only 48.38 % and 42.39 % of them had high awareness and positive perception towards measles vaccination respectively.

### 3.4 Timely uptake of second dose measles-containing vaccine

**Fig. 1:**
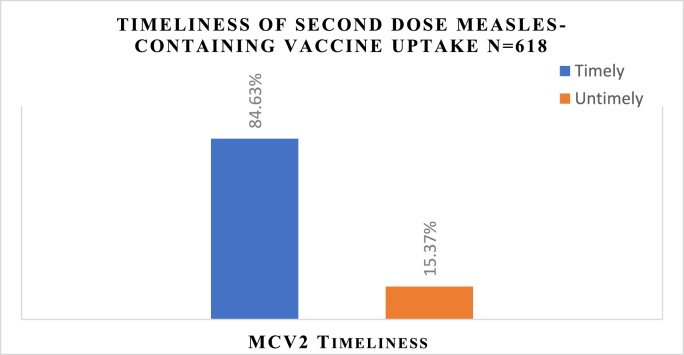
Timeliness of Second dose measles-containing vaccine uptake among children aged 24–36 months in Gondar City, Northwest Ethiopia, 2023.

Among the total of 618 children, 523 (84.63 %), (95 % CI: 81.77 %–87.48 %) were vaccinated for MCV2 timely (in the national recommended age) (Fig. 1). However, out of 618 children, forty-six (7.4 %) of children had no vaccination card during data collection time. For those children, the timeliness of MCV2 vaccination was checked by asking mothers about the age of their child when vaccinated.

### 3.5 MCV2 vaccination time for untimely vaccinated children

**Fig. 2:**
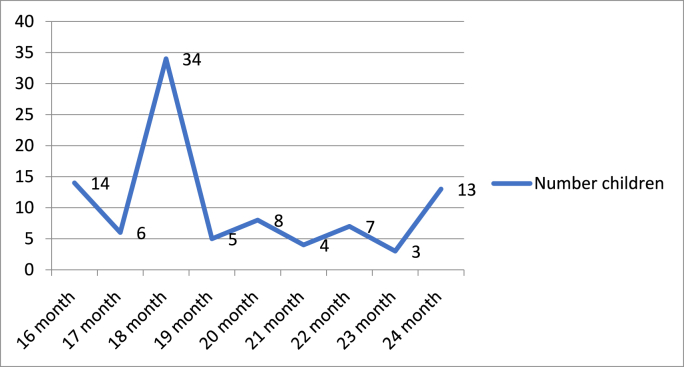
**MCV2** vaccination time for untimely vaccinated children.

Among the total of 95 untimely MCV2 vaccinated children, 34 (35.78 %) were vaccinated at 18 months of age or after 3 months of the nationally recommended age. The delay for untimely vaccinated children ranged from 1 month to 6 months (Fig. 2).

### 3.6 Factors associated with timely MCV2 uptake

After controlling the effect of potential confounding factors using multivariable logistic regression paternal educational status, ANC follow-up, Vitamin An uptake, Awareness, and perception of mothers to measles vaccination were statistically significant factors at a p-value of <0.05 with a 95 % confidence interval (Table 3).

**Table 3:** Factors associated with timely uptake MCV2 among children aged 24–36 months in Gondar City, Central Gondar, northwest Ethiopia, 2023. (N = 618).

Characteristics	Categories	MCV2 Timeliness		COR (95 % CI)	AOR (95 % CI)
		Timely	Untimely		
Age of Mothers	≤25	109 (88.62 %)	14 (11.38 %)	1	1
	26–30	160 (87.43 %	23 (12.57 %)	0.89 (0.44–1.81)	0.68 (0.30–1.54)
	31–35	151 (81.62 %)	34 (18.38 %)	0.57 (0.29–1.11)	0.44 (0.19–1.03)
	≥36	103 (81.10 %)	24 (18.90 %)	0.55 (0.27–1.12)	0.68 (0.25–1.81)
Maternal Educational status	Unable to read and write	44 (72.13 %)	17 (27.87 %)	1	1
	Primary level	132 (79.04 %)	35 (20.96 %)	1.45 (0.74–2.85)	0.36 (0.13–1.02)
	Secondary level	182 (84.26 %)	34 (15.74 %)	2.07 (1.05–4.03)	0.31 (0.09–1.03)
	College and above	165 (94.83 %)	9 (5.17 %)	7.08 (2.95–16.97)	0.32 (0.06–1.51)
Mother's Occupation	Housewife	281 (80.06 %)	70 (19.94 %)	1	1
	Farmer	10 (71.43 %)	4 (28.57 %)	0.62 (0.18–2.04)	1.32 (0.25–6.80)
	Privet Business	89 (85.58 %)	15 (14.42 %)	1.47 (0.81–2.71)	1.28 (0.58–2.81)
	Gov't Employee	143 (95.97 %)	6 (4.03 %)	5.93 (2.51–13.99)	3.07 (0.92–10.16)
Paternal educational status	Unable to read and write	8 (47.06 %)	9 (52.94 %)	0.87 (0.4–1.86)	1
	Primary level	200 (87.34 %)	29 (12.66 %)	7.75 (2.77–14.70)	2.25 (0.49–1.24)
	Secondary level	173 (87.82 %)	24 (12.18 %)	8.11 (2.85–16.02)	**3.83(1.35**–**5.46)∗**
	College and above	139 (81.29 %)	32 (18.71 %)	4.88 (1.75–13.64)	**5.84(1.55**–**8.18)∗∗**
Mass Media availability	Yes	457 (86.88 %)	69 (13.12 %)	2.60 (1.55–4.38)	0.85 (0.39–1.85)
	No	66 (71.74 %)	26 (28.26 %)	1	1
Average monthly income (US$)	<100	116 (76.82 %)	35 (23.18 %)	1	1
	100–200	225 (83.96 %)	43 (16.04 %)	1.57 (0.95–2.60)	1.14 (0.56–2.33)
	>200	182 (91.46 %)	17 (8.54 %)	3.23 (1.72–6.03)	1.11 (0.51–2.43)
Pregnancy status	Planned	405 (87.47 %)	58 (12.53 %)	2.18 (1.38–3.47)	1.26 (0.68–2.33)
	Unplanned	118 (76.13 %)	37 (23.87 %)	1	1
ANC follow up	No follow up	15 (65.22 %)	8 (34.78 %)	1	1
	1-3 follow-up	151 (80.32 %)	37 (19.68 %)	2.17 (0.85–5.51)	2.50 (0.72–8.76)
	≥4 follow up	357 (87.71 %)	50 (12.29 %)	3.80 (1.53–9.438)	**3.55(1.01**–**8.32)** ∗
TT vaccine status	Not received	97 (70.29 %)	41 (29.71 %)	1	1
	At least once	426 (88.75 %)	54 (11.25 %)	2.78 (2.10–5.29)	1.18 (0.62–2.25)
PNC service utilization	Not received	385 (81.91 %)	85 (18.09 %)	1	
	At least once	138 (93.24 %)	10 (6.76 %)	3.04 (1.53–6.03)	1.27 (0.58–2.81)
Place of vaccination	Hospital	209 (90.09 %)	23 (9.91 %)	3.02 (1.01–9.09)	1.38 (0.33–5.67)
	Health centers	288 (82.05 %)	63 (17.95 %)	1.52 (1.53–4.34)	0.73 (0.19–2.78)
	Health posts	11 (73.33 %)	4 (26.67 %)	0.91 (0.19–4.22)	0.49 (0.07–3.19)
	Outreach site	15 (75.00 %)	5 (25 %)	1	1
Vitamin An uptake	No received	19 (44.19 %)	24 (55.81 %)	1	1
	1 dose received	112 (76.71 %)	34 (23.29 %)	4.16 (2.03–8.49)	**2.82(1.19**–**6.71)** ∗
	At least 2 doses	392 (91.38 %)	37 (8.62 %)	9.25 (6.71–16.67)	**6.39(2.92**–**12.59)** ∗∗∗
Level of Awareness	Low	242 (75.86 %)	77 (24.14 %)	1	1
	High	281 (93.98 %)	18 (6.02 %)	4.96 (2.89–8.53)	**2.04(1.05**–**3.99)** ∗
Perception	Negative	270 (75.84 %)	86 (24.16 %)	1	1
	Positive	253 (96.56 %)	9 (3.44 %)	8.95 (4.41–18.17)	**4.81(2.13**–**10.86)** ∗∗∗

∗ P-value <0.05, ∗∗ p-value <0.01, ∗∗∗ p-value <0.005 bold: statistically significant variables.

AOR: Adjusted odds Ratio COR: crude Odds ratio.

Those children whose fathers attend secondary education were almost four times more likely to be vaccinated MCV2 timely as compared to those fathers who were unable to read and write (Adjusted odds ratio (AOR): 3.83, 95 % CI: 1.35–5.46). Moreover, fathers who attend college and above their education increase the chance of vaccinating their children for MCV2 timely by 5.84 times as compared to their counterparts (AOR: 5.84, 95 % CI: 1.55–8.18). The odds of timely vaccination for MCV2 were 3.55 times higher among children of mothers who had four or more ANC follow-ups (AOR: 3.55, 95 % CI: 1.01–8.32). Children who received one dose of vitamin A had 2.82 times higher odds, and those who received at least two doses had 6.39 times higher odds, of receiving MCV2 on time compared to those who did not receive any doses. Mothers with high awareness and positive perceptions of measles vaccination had 2 times and 4.8 times higher odds, respectively, of vaccinating their children on time for MCV2 compared to mothers with low awareness and poor perceptions.

## 4 Discussion

Timely measles vaccination is a vital public health intervention that offers protection against disease, safeguards vulnerable populations, prevents complications, and supports broader public health goals of disease control and eradication. This study assessed the timeliness of MCV2, hence, 84.63 % (95 % CI: 81.77 %–87.48 %) of children were vaccinated timely i.e. the nationally recommended time. The result was consistent with a study conducted in Debre Libanos district in the Oromia region, where 82.6 % of children had received MCV2 on time.^28^ However, the findings of this study were higher than a study conducted in Bangladesh (53 %), Tanzania (70 %), and China (77 %).^10^^,^^15^^,^^29^ This discrepancy may be due to the time of the study conducted. Moreover, the government of Ethiopia with the collaboration of non-governmental organizations had made a remarkable effort to increase measles vaccination coverage in the past few years.

The result of this study revealed that fathers with higher levels of education have a higher chance of vaccinating their children for MCV2 timely. The finding is consistent with the other studies.^30^^,^^31^ This is because fathers with the highest levels of education tend to have greater health literacy, which includes knowledge about the importance of vaccinations and adherence to vaccination schedules. They may better understand the risks of vaccine-preventable diseases like measles and the benefits of vaccination for their children. Fathers with higher education levels may be more proactive in seeking preventive healthcare for their children, including vaccinations. They may engage more readily with healthcare providers and follow recommended vaccination schedules. Fathers with a higher education level may correlate with greater involvement in healthcare decisions and a stronger commitment to preventive health measures like vaccination.

Children who took vitamin A in one or more doses have higher odds of vaccinating MCV2 timely than children who had not received any dose. The result is supported by a study conducted in Kenya.^32^ The possible justification may be that repeated encounters with the healthcare system for Vitamin A supplementation may provide opportunities for children also to receive essential health education, including vaccination schedules. Thus, children who receive repeated Vitamin A vaccinations may be more likely also to receive timely vaccinations, including the second dose of the measles vaccine. This result implies that providing health education during each visit for mothers/caregivers may help to remember the schedule and vaccinate their children accordingly.

The finding showed that the odds of timely vaccination of MCV2 were higher among children of mothers who had four or more ANC visits than those who hadn't had any visits. The finding is supported by studies.^33^^,^^34^ This is the fact that repeated ANC visits often indicate that mothers are more engaged with healthcare services. This heightened awareness and interaction with healthcare providers may also extend to ensuring timely vaccinations for their children, including the second dose of the measles vaccine. Also, ANC visits are opportunities for healthcare providers to educate mothers about the importance of childhood vaccinations, including the recommended schedules of each vaccine. Mothers who attend ANC visits regularly are more likely to receive this education and subsequently ensure their children receive timely vaccinations including MCV2. This result suggests that strengthening maternal health services has a greater effect on the timely administration of measles vaccination.

Mothers who had high awareness about measles vaccination were two times more likely to vaccinate their children timely for MCV2 than mothers who had low awareness. This study is supported by other studies.^10^^,^^13^ This is the fact that mothers who are well-informed about the importance of measles vaccination are more likely to ensure that their children receive both doses at the recommended time. Awareness campaigns, education programs, and access to reliable healthcare information can influence maternal knowledge and behavior regarding vaccination. Similarly, mothers who had a positive perception of measles vaccination were more likely to vaccinate their children for MCV2 timely as compared to mothers who had a negative perception. A Nigerian study supports this finding.^35^ This is because mothers who perceive measles vaccination positively are more likely to trust its efficacy and safety profile in protecting their children from measles and its complications. This trust can motivate them to ensure their children receive both doses of the vaccine on time, as recommended by healthcare authorities. Moreover, mothers who have a positive perception of measles vaccination may feel a strong sense of responsibility for their child's health and well-being. This sense of responsibility can drive them to ensure that their children receive all recommended vaccinations, including the second dose of the measles vaccine, promptly. Effective communication from healthcare providers plays a crucial role in shaping mothers' awareness and perceptions of measles vaccination. Positive interactions with healthcare professionals who provide accurate information, address concerns, and emphasize the importance of vaccination can strengthen mothers' confidence in vaccination and support timely vaccination practices.

## 5 Limitations of the study

Since the study relies on vaccination cards and verbal accounts from mothers/caregivers, the latter may be subject to recall bias. However, only 7 % of children had no vaccination card, suggesting that the degree of bias may not be significant. Secondly, this study focuses solely on children who received the second dose of the measles-containing vaccine. Although we aimed to minimize recall bias by including children aged 24–36 months, this design restricts our understanding of overall vaccination coverage in the population. Additionally, as a cross-sectional study, only correlations—not causations—can be established.

## 6 Conclusion and recommendations

A remarkable effort has been made in the timely administration of MCV2 for children in the study area. Due to this, among MCV2 vaccinated children around 85 % of them took the vaccine timely (on nationally recommended time). Paternal educational status, ANC follow-up, vitamin An uptake, awareness, and perception of mothers to measles vaccination were statistically significant determinants for timely uptake of MCV2. Since strengthening maternal and child health services affects timely childhood measles vaccination, efforts to increase service uptake should be continued in a better way by healthcare workers and health authorities. Healthcare providers should work on increasing the awareness and perception of mothers about measles vaccination. Health authorities should incorporate the timeliness of measles vaccinations into national health policies. Their focus should not only be on coverage but also on ensuring timely vaccinations to improve vaccine performance. Further research should be done on measles vaccine timeliness in another setting.

## Ethical consideration

Ethical clearance was obtained from the ethical review committee of the School of Nursing on behalf of the Institutional Review Board (IRB) of the University of Gondar with ethical approval number S/N/164/2015, and a letter of permission was obtained from the Gondar city health office. Before the interview, each participant provided written informed consent after being explained the purpose of the study. All information gathered in the study was kept confidential.

## Funding

None.

## Declaration of competing interest

The author hereby declares to Journal of virus eradication is that there are no conflicts of interest related to the publication of this research.

## Figures and Tables

**Table:** 

ANC	Antenatal Care
BCG	Bacilli Calmette-Guerin
MCV1	The first dose of Measles-Containing Vaccine
MCV2	Second dose of Measles-Containing Vaccine
PNC	Postnatal Care
TT	Tetanus Toxoid
WHO	World Health Organization

## Data Availability

Data will be made available on request.

## References

[1] Berche P. (2022). History of measles. Press.

[2] World Health Organization (2022).

[3] Patel M.K., Goodson J.L., Alexander J.P., Kretsinger K., Sodha S.V., Steulet C. (2020). Progress toward regional measles elimination — worldwide , 2000 – 2019.

[4] WHO Press (2013).

[5] CDC (2020). Progress toward regional measles elimination — worldwide, 2000–2020. https://www.cdc.gov/mmwr/volumes/70/wr/mm7045a1.htm.

[6] (2019). WHO. position paper. https://www.afro.who.int/news/ethiopia-launches-measles-vaccine-second-dose-mcv2-introduction.

[7] World Health Organization (21 April 2022).

[8] WHO. WHO Recommendations for Routine Immunization: A User ‘ S Guide to the Summary Tables.

[9] Kroger A, Bahta L, Long S SP. General best practice guidelines for immunization. Best Practices Guidance of the Advisory Committee on Immunization Practices (ACIP).

[10] Jaffar H., Mbb O. (2021). Tanzania.

[11] Hu Y., Wang Y., Chen Y., Liang H., Chen Z. (2018). Measles vaccination coverage , determinants of delayed vaccination and reasons for non-vaccination among children aged 24 – 35 months in Zhejiang province. China (Q Forecast Rep).

[12] Mekonnen Z.A., Were M.C., Tilahun B. (2020). BMC Public Health.

[13] Marefiaw T.A., Yenesew M.A., Mihirete K.M. (2019). Age-appropriate vaccination coverage and its associated factors for pentavalent 1-3 and measles vaccine doses, in northeast Ethiopia: a community-based cross-sectional study. PLoS One.

[14] Hu Y., Li Q., Luo S., Lou L., Qi X., Xie S. (2013). Timeliness vaccination of measles containing vaccine and barriers to vaccination among Migrant children in East China.

[15] Sheikh N., Sultana M., Ali N. (2018). Coverage, Timelines, and determinants of incomplete immunization in Bangladesh. Trav Med Infect Dis.

[16] Qu Shujuan, Min Zhou K.S.C., Wh (2024). Predictors of parental acceptance to live attenuated influenza vaccine for children. Hum Vaccines Immunother [Internet].

[17] WHO/UNICEF (2022).

[18] Tadesse A.W., Sahlu D., Benayew M. (2022). Second-dose measles vaccination and associated factors among under-five children in urban areas of North Shoa Zone, Central Ethiopia, 2022. Front Public Health.

[19] Demewoz A., Wubie M., Mengie M.G. (2023).

[20] Adisu M.A., Bogale W.A., Alemu T.G. (2024 May 2). Second dose of measles-containing vaccine coverage and associated factors among children aged 24–36 months in Gondar city, Central Gondar, Northwest Ethiopia, 2023. Front Public Health.

[21] CDC (2022). Worlds measles outbreak report. https://www.cdc.gov/globalhealth/measles/data/global-measles-outbreaks.html.

[22] Smetana J., Chlibek R., Hák Z. (2018). Serological response to measles revaccination. Epidemiol Infect.

[23] Gondar city Health Department (2023).

[24] Ministry of Health Ethiopia (2021).

[25] Grinberg K., Sela Y. (2021).

[26] Goshu A., Woldemariam M., Tigabu B., Getie M., Molla G., Animut Y. (2022). Vaccine : X Less than one-fifth of Ethiopian children were vaccinated for measles second dose ; evidence from the Ethiopian mini demographic and health survey 2019. Vaccine X [Internet].

[27] Teshale A.B., Amare T. (2023). Exploring spatial variations and the individual and contextual factors of uptake of measles-containing second dose vaccine among children aged 24 to 35 months in Ethiopia. PLoS One.

[28] Dejene H., Girma D., Geleta L.A., Legesse E. (2022 Jul 27). Vaccination timeliness and associated factors among children aged 12–23 months in Debre Libanos district of north Shewa zone, Oromia regional state, Ethiopia. Frontiers in Pediatrics.

[29] Tang X., Geater A., Mcneil E., Zhou H., Deng Q. (2017). Timeliness and completeness of measles vaccination among children in rural areas of Guangxi , China : a strati fi ed three-stage cluster survey. J Epidemiol.

[30] Barrow A., Afape A.O., Cham D., Azubuike P.C. (2023). Uptake and determinants of childhood vaccination status among children aged 0–12 months in three West African countries. BMC Public Health.

[31] Nour T.Y., Farah A.M., Ali O.M., Osman M.O. (2020).

[32] Makokha F.M., Wanjala P.M., Githuku J., Kutima H.L. (2015). Uptake of second dose of measles-containing vaccine among children in Kakamega county , Kenya. Int J Sci Res Publ. Int J Sci Res Publ [Internet].

[33] Fenta S.M., Biresaw H.B., Fentaw K.D., Gebremichael S.G. (2021). Determinants of full childhood immunization among children aged 12–23 months in sub-Saharan Africa: a multilevel analysis using Demographic and Health Survey Data. Trop Med Health.

[34] Krishnamoorthy Y., Rehman T. (2022). Impact of antenatal care visits on childhood immunization: a propensity score-matched analysis using nationally representative survey. Fam Pract.

[35] Oluseye O.M., Jimoh N.A., Ogunleye C.A. (2021). Knowledge and attitude of mothers towards measles and measles, mumps and Rubella (Mmr) vaccine in Idi- Aba community Abeokuta, Nigeria. Open J Med Res (ISSN 2734-2093).

