# Supplementary material for: Timeliness of the second dose of measles-containing vaccine uptake and its determinants among children aged 24–36 months in Gondar City, Northwest Ethiopia, 2023: Community-based cross-sectional study design
Source: J Virus Erad. 2025 Mar 17;11(2):100594. doi: 10.1016/j.jve.2025.100594 (PMC11987597; doi:10.1016/j.jve.2025.100594)
Supplement: Multimedia component 1 [file mmc1.docx]

11 rural kebeles

25 urban kebeles

**Aba Antonius 73**

**Fenter 139**

**Loza**

**129**

**Keha Eyesus 232**

**AbaSamuel 808**

**Arbegnoch**

239

**Samuna ber 335**

**Ayer Tena 483**

Lideta

1348

13

22

23

39

136

40

56

81

224

**Supplemental figure 1:** Schematic presentation of sampling procedure to study Timeliness of second dose measles vaccination uptake and its determinants among children aged 24–36 months in Gondar city, Northwest Ethiopia, 2023: A community based cross-sectional study
